# The Synthetic β-Nitrostyrene Derivative CYT-Rx20 Inhibits Esophageal Tumor Growth and Metastasis via PI3K/AKT and STAT3 Pathways

**DOI:** 10.1371/journal.pone.0166453

**Published:** 2016-11-22

**Authors:** Wen-Chin Chiu, Yi-Chen Lee, Yu-Han Su, Yen-Yun Wang, Chun-Hao Tsai, Yi-An Hou, Chie-Hong Wang, Ying-Fong Huang, Chih-Jen Huang, Shah-Hwa Chou, Pei-Wen Hsieh, Shyng-Shiou F. Yuan

**Affiliations:** 1 Division of Thoracic Surgery, Department of Surgery, Kaohsiung Medical University Hospital, Kaohsiung Medical University, Kaohsiung, Taiwan; 2 Department of Anatomy, School of Medicine, College of Medicine, Kaohsiung Medical University, Kaohsiung, Taiwan; 3 Translational Research Center, Kaohsiung Medical University Hospital, Kaohsiung Medical University, Kaohsiung, Taiwan; 4 Department of Medical Research, Kaohsiung Medical University Hospital, Kaohsiung Medical University, Kaohsiung, Taiwan; 5 Graduate Institute of Medicine, College of Medicine, Kaohsiung Medical University, Kaohsiung, Taiwan; 6 Department of Respiratory Therapy, College of Medicine, Kaohsiung Medical University, Kaohsiung, Taiwan; 7 Department of Nuclear Medicine, Kaohsiung Medical University Hospital, Kaohsiung Medical University, Kaohsiung, Taiwan; 8 Department of Radiation Oncology, Kaohsiung Medical University Hospital, Kaohsiung Medical University, Kaohsiung, Taiwan; 9 Graduate Institute of Natural Products, School of Traditional Chinese Medicine and Graduate Institute of Biomedical Sciences, College of Medicine, Chang Gung University, Taoyuan, Taiwan; 10 Department of Anesthesiology, Chang Gung Memorial Hospital, Taoyuan, Taiwan; 11 Department of Obstetrics and Gynecology, Kaohsiung Medical University Hospital, Kaohsiung Medical University, Kaohsiung, Taiwan; University of South Alabama Mitchell Cancer Institute, UNITED STATES

## Abstract

The β-nitrostyrene family have been implicated for anti-cancer property. However, the pharmacological role of β-nitrostyrene in esophageal cancer remain unclear. Here, a β-nitrostyrene derivative, CYT-Rx20, was synthesized and assessed for its anti-cancer activities and underlying mechanism in esophageal cancer. CYT-Rx20 induced cytotoxicity in esophageal cancer cells by promoting apoptosis through activation of caspase cascade and poly(ADP-ribose) polymerase (PARP) cleavage. Besides, CYT-Rx20 inhibited esophageal cancer cell migration and invasion by regulating the expression of epithelial to mesenchymal transition (EMT) markers. CYT-Rx20 decreased cell viability and migration through suppression of the PI3K/AKT and STAT3 pathways. Of note, the cytotoxicity and anti-migratory effect of CYT-Rx20 were enhanced by co-treatment with SC79 (AKT activator) or colivelin (STAT3 activator), suggesting the dependency of esophageal cancer cells on AKT and STAT3 for survival and migration, an oncogene addiction phenomenon. In xenograft tumor-bearing mice, CYT-Rx20 significantly reduced tumor growth of the implanted esophageal cancer cells accompanied by decreased Ki-67, phospho-AKT, and phospho-STAT3 expression. In orthotopic esophageal cancer mouse model, decreased tumor growth and lung metastasis with reduced Ki-67 and phospho-STAT3 expression were observed in mice treated with CYT-Rx20. Together, our results suggest that CYT-Rx20 is a potential β-nitrostyrene-based anticancer compound against the tumor growth and metastasis of esophageal cancer.

## Introduction

Esophageal cancer is the eighth most common malignancies worldwide including eastern Asia with the overall five year survival rate ranging from 15% to 25% [1–3]. Esophageal squamous cell carcinoma (ESCC) and esophageal adenocarcinoma (EAC) are the two major subtypes of esophageal cancer with ESCC accounting for more than 90% of esophageal cancer cases and characterized by frequent relapses and metastatic capabilities [4]. Despite advance in early detection and treatment with concurrent chemoradiation therapy (CCRT) alone or as an adjunct to surgery [5], esophageal cancer remains a major challenge and requires more effective therapeutic strategies.

The β-nitrostyrene family compounds have been recognized for anti-cancer, anti-microbial, anti-inflammatory, and anti-platelet activities [6–11]. β-nitrostyrene derivatives induced pro-apoptotic effect through inhibition of protein tyrosine phosphatases (PTPs) and phosphatases 2A (PP2A) [12,13] and inhibited the growth of cancer cells derived from stomach and immune responses of macrophages [6]. In addition, they induced the apoptosis in Burkitt's lymphoma derived cells via chromatin condensation and membrane blebbing [14]. The potent effects of β-nitrostyrene derivatives on inhibiting the TNFα-induced NF-κB survival pathway in a truncated retinoid X receptor α (tRXRα)-dependent manner resulted in a synergistic effect of β-nitrostyrene derivatives and TNFα on inducing breast cancer cell apoptosis [15]. Moreover, two β-nitrostyrene derivative compounds NTS1 and NTS2 induced apoptosis through cytochrome c release from mitochondria to cytosol and caspase activation in Ehrlich ascitic tumor cells [16]. Recently, the inhibitory effect of 3,4-methylenedioxy-β-nitrostyrene on breast cancer cell adhesion and migration was demonstrated by suppressing β1 integrin and surface protein disulfide isomerase [17].

Our previous studies demonstrated that CYT-Rx20 (3′-hydroxy-4′-methoxy-β- methyl-β-nitrostyrene), a synthetic derivative of β-nitrostyrene, tended to increase the anti-platelet activity and induces breast cancer cell death and autophagy through ROS-mediated MEK/ERK pathway [10,18]. However, the pharmacological effects and molecular mechanism of CYT-Rx20’s action in esophageal cancer remain unknown. In this study, the anti-esophageal cancer activity of CYT-Rx20 is explored by studying its biological effects and the underlying mechanisms in vitro and in vivo.

## Materials and Methods

### Materials

CYT-Rx20 was synthesized by methods that have been previously described [11]. Chemotherapeutic agents used in this study included 5-Fluorouracil (5-FU) (Mayne Pharma Pty Ltd, Mulgrave, Victoria, Australia). SC79 was purchased from Tocris Biosciences (Bristol, UK). Colivelin was purchased from Santa Cruz Biotechnology (Santa Cruz, CA). Antibodies recognizing AKT, phospho-STAT3 (Tyr705), caspase 9, Cdc25C, and cleaved poly(ADP-ribose) polymerase (PARP) (Asp214) were obtained from Cell Signaling Technology (Danvers, MA). Cyclin B1, phospho-p85 (Tyr 467), phospho-Akt (Ser473), p85, STAT3, ZO-1, ZEB1, Snail, and β-actin antibodies were purchased from GeneTex (Irvin, CA). Slug antibody was obtained from ThermoFisher (Wilmington, DE). Caspase 3 and caspase 8 antibodies were purchased from Novus (Irvin, CA). Antibodies against phospho-p21 (Thr145), N-cadherin and α-tubulin were obtained from Abcam (Cambridge, MA). Ki-67 antibody was obtained from Biorbyt (Riverside, UK). Other chemicals were obtained from Sigma (St. Louis, MO).

### Cell Culture

The esophageal cancer cell line (KYSE70) was kindly provided by Dr. Yi-Ching Wang (Department of Pharmacology, National Cheng Kung University, Tainan, Taiwan). Human ESCC cell line (TE8) was kindly provided by Dr. Mien-Chie Hung (MD Anderson Cancer Center, Houston, TX). Normal human esophageal squamous cell line (HET-1A) was kindly provided by Dr. Pei-Jung Lu (Institute of Clinical Medicine, National Cheng Kung University, Tainan, Taiwan). KYSE70 was cultured in RPMI1640 medium (Invitrogen, Carlsband, CA). TE8 cells were cultured in DMEM/F12 medium (Invitrogen, Carlsband, CA). The HET-1A cell line was cultured in BEBM (Bronchial/Tracheal Epithelial Cell Basal Medium; CELL Applications, San Diego, CA). All the medium were supplemented with 10% (v/v) fetal bovine serum, 100 U/mL penicillin, 100 μg/mL streptomycin, and 2.5 μg/mL amphotericin B (Biological Industries, Haemek, Israel). All cells were grown in a 5% CO_2_ incubator at 37°C.

### XTT colorimetric assay

Cell viability of KYSE70 and TE8 cells treated with CYT-Rx20 was determined by tetrazolium salt 2,3-bis[2-methoxy-4-nitro-5-sulfophenyl]-2H-tetrazolium-5- carboxanilide (XTT) assay (Roche Applied Science, Indianapolis, IN). Cells were seeded at the density of 5x10^3^ cells/well in 96-well plates and allowed to grow for additional times. The cell culture medium was removed and XTT assay was carried out by the methods previously described [19]. Three independent experiments with four replicates in each were performed.

### Annexin V/PI apoptosis assay

Annexin V/PI apoptosis detection kit was purchased from BD Biosciences (Franklin Lakes, NJ) and detailed procedure was followed according to the manufacturer's instructions. Briefly, after treatment with CYT-Rx20 for 48 h, the cells were collected and washed twice with cold PBS and then adjusted to 5×10^5^ cells/500 μl in binding buffer containing annexin V-FITC (1 μg/ml) and PI before analysis by flow cytometry (BD Biosciences).

### Terminal deoxynucleotidyl transferase dUTP nick-end labeling (TUNEL) assay

Cells were treated with the indicated concentrations of CYT-Rx20 for 48 h and then stained for apoptotic cells using the DeadEnd Colorimetric TUNEL system from Promega (Madison, WI). The procedures were followed according to a previous described report [20].

### Fluorescence-activated cell sorting (FACS) analysis

FACS analysis was applied to analyze the cell cycle distribution. KYSE70 and TE8 cells were treated with CYT-Rx20 and plated on six-well plates (Corning Life Sciences, Corning, NY). Cells were trypsinized by 1% trypsin-EDTA (Invitrogen, Carlsband, CA), fixed in 75% ethanol (Sigma, St. Louis, MO) and stored at -20°C for at least 16 h. Before analysis, cells were stained for 30 min at 37°C with PBS containing 50 μg/mL propidium iodide and 4 kU/mL RNase (BD Biosciences, San Jose, CA). The DNA contents were measured by FACScan flow cytometer (BD Biosciences, Franklin Lakes, NJ) and cell cycle distribution was calculated using Winmdi 2.8 software (J. Trotter, Scripps Research Institute, La Jolla, CA).

### Immunoblotting analysis

The whole cell extracts were collected by lysing cells in the buffer containing 50 mM Hepes, 6 mM EDTA, and 1% Triton X-100 supplemented with phosphatase inhibitor (Sigma, St. Louis, MO) and complete protease inhibitor cocktail (Roche Applied Science, Indianapolis, IN). Total protein was separated by centrifugation at 12000g for 20 min at 4°C. The protein concentration was determined using the Bradford assay (Bio-Rad, Hercules, CA) with BSA as a standard. Equal amounts of proteins were subjected to 10% SDS-PAGE and transferred onto nitrocellulose membranes (Pall corporation, East Hills, NY). The immunoblot analysis for protein expression was performed as described previously [19]. The chemiluminescent signal was captured by a ChemiDoc^TM^ XRS+ System (Bio-Rad Laboratories, Hercules, CA) and quantified with Image Lab software (Bio-Rad Laboratories, Hercules, CA).

### Cell migration and invasion assays

Cell migration was estimated by a modified Boyden chamber assay. Cells (5×10^4^ cells/well) suspended in 2% FBS medium were placed in the upper chamber of 8-μm pore size transwells (24-well, Corning Life Sciences, Corning, NY), and medium with 10% FBS was added to the lower chamber. After incubation for 48 hours, the unmigrated cells were removed from the upper surface of the membrane, and the migrated cells on the lower surface of the membrane were fixed in 100% methanol and stained with Giemsa stain (Sigma, St. Louis, MO). The cell migration was determined by counting the number of the migrated cells under a microscope at ×100 magnification. Four visual fields were chosen randomly and the average area of migrated cells in the four fields was calculated for each group. In vitro cell invasion assay was determined using transwells with 8 μm pores coated with Matrigel (Corning Life Sciences, Corning, NY) with the same protocol of cell migration as described above.

### Anchorage-independent soft agar assay

Soft agar colony formation assay was performed according to the previous study [19]. Briefly, medium mixed with 0.5% agar (Sigma) was paved onto a 24-well plate (Corning Life Sciences, Corning, NY) and allowed to solidify. The dishes were overlaid with 5x10^3^ CYT-Rx20-treated KYSE70 cells in medium with 5% fetal bovine serum and 0.3% agarose. Cultures were allowed to form colonies for 28 days and replenished with medium every 3–4 days. Colonies were stained with 0.005% crystal violet (Sigma, St. Louis, MO) and then were counted using a dissecting microscope (Nikon, Tokyo, Japan).

### In vivo tumor xenograft study

All experiments involving mice were performed according to the guidelines of the Animal Committee and approved by the Institutional Animal Care and Use Committee (IACUC no. 102009) of Kaohsiung Medical University, Taiwan. Six-week-old female immunodeficient BALB/cAnN.Cg-*Foxn1*^*nu*^/CrlNarl mice, obtained from the National Laboratory Animal Center of Taiwan, were used in this study. The mice were housed 2~3 animals per cage with sterilized stainless steel cover and bedding, under 12 h light/dark cycle, 22 ± 2°C room temperature, and 40–60% relative humidity. Food and drinking water were provided *ad libitum*. The mice were then subcutaneously injected with 5 × 10^6^ KYSE70 cells at the right and left flank. When tumors became visible (approximately an average diameter of 3 mm), the mice were intraperitoneally injected with CYT-Rx20, dissolved in DMSO and then diluted with 100 μL normal saline (0.1% DMSO), at 5 μg/g body weight or 25 μg/g body weight three times a week (n = 10 for each group). The control mice were injected with 0.1% DMSO in normal saline only. Tumor sizes were measured by using calipers every week and tumor volumes were calculated according to a standard formula: (width^2^ x length)/2. After 4 weeks, the mice were sacrificed by deep anesthesia with isoflurane (Baxter, Deerfield, IL) and, after tumor weight was measured, tumor and mouse organs were harvested for histological examination.

### Orthotopic esophageal cancer mouse model

Six-week-old female immunodeficient BALB/cAnN.Cg-*Foxn1*^*nu*^/CrlNarl mice were obtained from the National Laboratory Animal Center of Taiwan. The mice were housed 2~3 animals per cage in pathogen free conditions, under 12 h light/dark cycle, 22 ± 2°C room temperature, and 40–60% relative humidity with *ad libitum* food and drinking water. Orthotopic esophageal cancer model was performed according to a previous study [21]. A skin incision was made in the middle of the upper abdomen from the xiphoid process. The liver was raised up, and then the stomach was pulled down so that the abdominal esophagus was seen. A syringe with a 31G needle was inserted into the abdominal esophagus and injected with 1 × 10^6^ luciferase-expressing KYSE70 cells. Then, the mice were intraperitoneally injected with either 0 μg/g or 5 μg/g of CYT-Rx20 dissolved in DMSO with 100 μl normal saline (0.1% DMSO) three times a week (n = 4–5 for each group). At 4 weeks after tumor inoculation, the mice were analyzed for the presence of bioluminescent signals by Xenogen IVIS Spectrum in vivo imaging system (Caliper Life Sciences, Hopkinton, MA). The mice were anesthetized with isoflurane (Baxter, Deerfield, IL) using a XGI-8 Gas Anesthesia System (Caliper Life Sciences, Hopkinton, MA) followed by intraperitoneal injection of D-luciferin (150 mg/kg; PerkinElmer, Waltham, MA) for the detection of luciferase expression. The optical images were acquired and analyzed by Xenogen Living Image software (Caliper Life Sciences, Hopkinton, MA). Then, the mice were sacrificed by deep anesthesia with isoflurane (Baxter, Deerfield, IL), and its main esophageal tumor and potentially metastatic organs were harvested for histological examination. The animal study was approved by the Institutional Animal Care and Use Committee (IACUC No. 102009) of Kaohsiung Medical University, Taiwan.

### Immunohistochemical Analysis

Immunohistochemical (IHC) staining for Ki-67, phospho-AKT, phospho-STAT3, and cleaved caspase-3 was performed on a Leica Bond-Max autostainer (Leica Microsystems, Bannockburn, IL). Sections cut from formalin-fixed, paraffin-embedded tissue blocks were baked at 60°C for 1 h, then deparaffinized in Bond Dewax solution at 72°C for 30 min and rehydrated in Bond Wash solution. Heat-induced antigen retrieval was carried out with Bond Epitope Retrieval solution 2 for 20 min at 100°C and peroxide block placement on the slides for 5 min at room temperature. Slides were then incubated with antibodies at a dilution of 1:200–500 for 30 min and Post Primary reagent for 8 min at room temperature. Antibody detection was carried out with the Bond Polymer placement for 8 min and color development with DAB (3,3’-diaminobenzidine tetrahydrochloride) as a chromogen for 5 min at room temperature. Slides were counterstained with hematoxylin for 5 min, followed by mounting of the slides and then the images of IHC-stained sections were captured by a Nikon E-800M microscope (Tokyo, Japan) and processed by Nikon NIS-Elements Version 4.30 software (Tokyo, Japan). The results for Ki-67, phospho-AKT, and phospho-STAT3 staining were determined by two independent experts under the same condition. For quantification, the sections of each immunostaining were scored using the method of histochemical score (H-score), which was calculated as the product of percentage of stained cells and intensity of staining [22,23].

### Statistical analysis

Data are given as means ± S.E.M. and all experiments were repeated at least three times independently. All statistical analyses were performed using the SPSS 20.0 statistical software for PC (SPSS, Chicago, IL) and *P* values less than 0.05 were considered statistically significant. Statistical analysis between the control and CYT-Rx20 group was performed using two-sided Student’s *t*-test. One-way ANOVA, combined with Tukey’s multiple-comparison test, was used to evaluate the statistical significance of differences between three or more groups.

## Results

### The synthetic β-nitrostyrene derivative CYT-Rx20 induced esophageal cancer cell death

**Fig 1:**
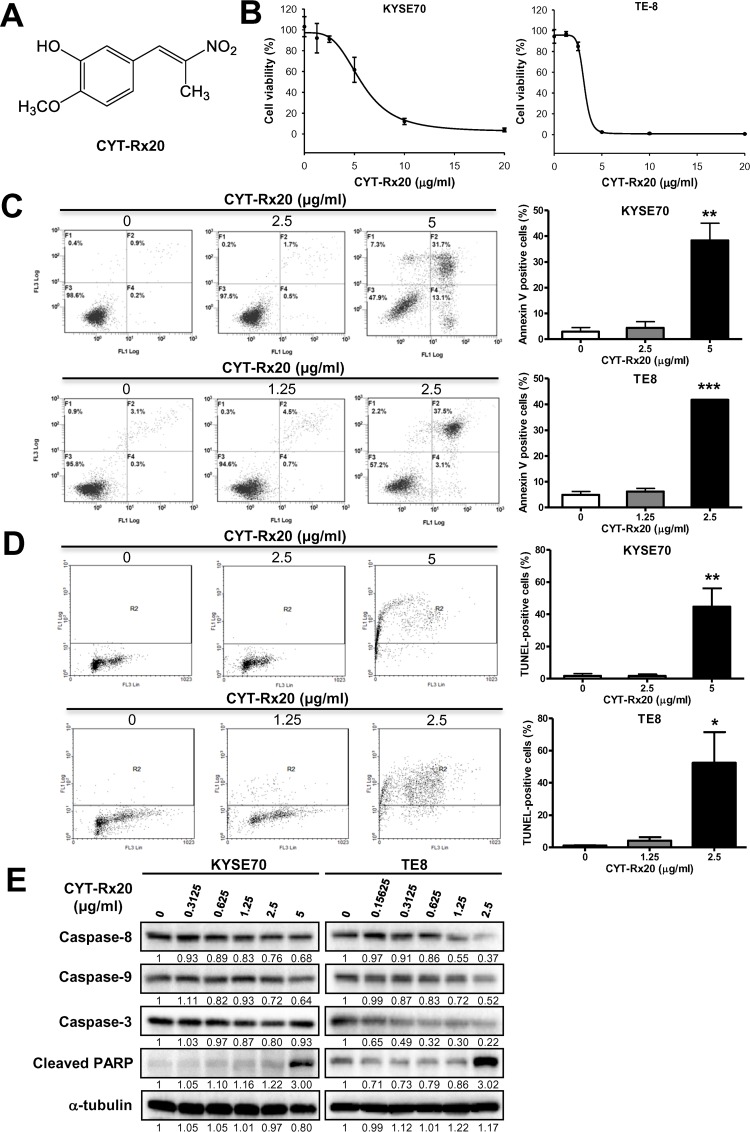
Effect of CYT-Rx20 on cytotoxicity and cell apoptosis in esophageal cancer cells. (A) Chemical structure of CYT-Rx20. (B) Effects of CYT-Rx20 on KYSE70 and TE8 cell viability. KYSE70 and TE8 cells were treated with various concentrations of CYT-Rx20 for 48 h and assessed by XTT colorimetric assay. The representative results are repeated at least three times and five replicate wells per CYT-Rx20 concentration in each experiment. (C) KYSE70 and TE8 cells were treated with CYT-Rx20 for 48 h, and cell death was examined with Annexin V/PI staining followed by flow cytometric analysis. (D) Effect of CYT-Rx20 on KYSE70 and TE8 cell apoptosis. TUNEL staining of cells with CYT-Rx20 was analyzed at 48 hours. One-thousand cells were counted for positivity for each stain. (E) Caspase-associated proteins were analyzed by immunoblotting after KYSE70 and TE8 cells were treated with the indicated concentrations of CYT-Rx20 for 24 h. The expression of α-tubulin was used as the internal control. The representative results are from three separate experiments. Results are means ± SEM (n = 3–5). *P < 0.05, **P <0.01, ***P <0.001 compared with the control group.

The cytotoxicity of a β-nitrostyrene derivative, CYT-Rx20 (Fig 1A), on human esophageal cancer cells was analyzed by XTT assay. CYT-Rx20 exhibited the potent cytotoxic effect against esophageal cancer cells (KYSE70 and TE8, Fig 1B) in comparison with the HET-1A normal esophageal squamous cell line (S1 Fig). The inhibitory concentrations of IC50 after 48 h treatment with CYT-Rx20 on two esophageal cancer cell lines, KYSE70 and TE8, were 5.16 ± 0.21 and 3.07 ± 0.04 μg/ml, respectively (Table 1), compared to the HET-1A normal esophageal cells (6.05 ± 2.35 μg/ml). CYT-Rx20 showed a higher potent of cytotoxicity on esophageal cancer cells than the clinically used drug, 5-Fu (Table 1). Next we analyzed cell apoptosis by Annexin V/PI staining after CYT-Rx20 treatment for 48 h. We observed that apoptosis was statistically significantly higher in CYT-Rx20-treated KYSE70 and TE8 cells compared with untreated control cells (KYSE70: 38.4 ± 6.7% vs. 2.9 ± 1.6%, *P* <0.01; TE8: 41.8 ± 0.2% vs. 5.0 ± 1.3%, *P* <0.001) (Fig 1C). The apoptotic cell death induced by CYT-Rx20 was further evidenced by TUNEL staining (KYSE70: 44.7 ± 11.4% vs. 1.7 ± 1.5%, *P* <0.01; TE8: 52.4 ± 19.1% vs. 1.2 ± 0.3%, *P* <0.05) (Fig 1D). Further, CYT-Rx20 treatment decreased the protein expression of pro-caspase-8, 9, 3, and increased the levels of cleaved PARP (Fig 1E).

**Table 1:** Cytotoxicity^a^ of CYT-Rx20 on esophageal cancer cell lines.

Cell type	Cell lines	CYT-Rx20 IC50^b^ (μg/ml)	5-FU IC50^b^ (μg/ml)
Esophageal cancer	KYSE70	5.16 ± 0.21	11.71 ± 2.14
	TE8	3.07 ± 0.04	15.45 ± 1.45

^a^The cells were treated with CYT-Rx20 at different concentrations for 48 h, before proceeding with the XTT assay.

^b^Means ± SEM of three independent experiments.

### Effect of CYT-Rx20 on esophageal cancer cell migration and invasion in vitro

**Fig 2:**
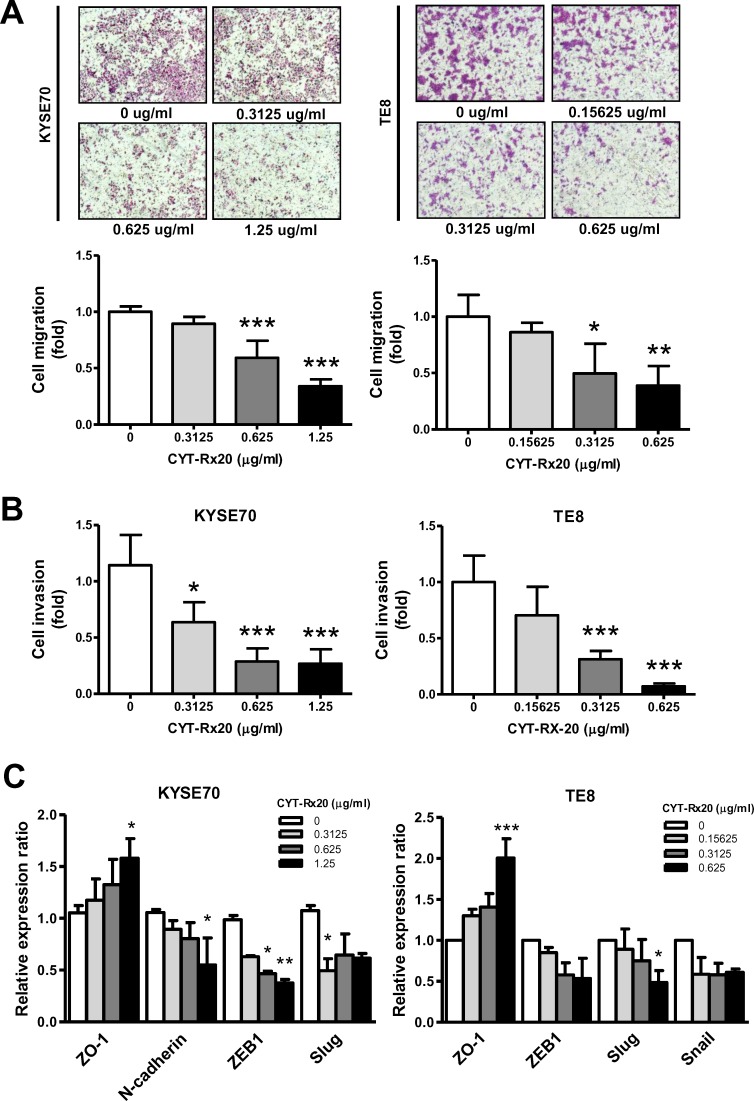
Effect of CYT-Rx20 on KYSE70 and TE8 cell migration and invasion. (A) The effect of CYT-Rx20 on cell migration was detected by using a modified Boyden chamber assay, as described in the Materials and methods section. The representative photographs are shown with ×40 magnification. Histograms represent quantification of cell migration and are expressed as the average area of the migrated cells in four fields of the group. (B) Effect of CYT-Rx20 on KYSE70 and TE8 cell invasion. Invasion of cells through transwell inserts containing Matrigel-coated membranes was assessed 48 h after CYT-Rx20 treatment. (C) Epithelial to mesenchymal transition (EMT) proteins were analyzed by immunoblotting after KYSE70 and TE8 cells were treated with the indicated concentrations of CYT-Rx20 for 24 h. The intensity of the protein band normalized to the internal control β-actin was calculated as the fold of controls (set as 1) and then depicted as histograms.

Directed migration of cells in vitro, measured in a modified Boyden chamber assay as the area of migrated cells after CYT-Rx20 treatment for 48 h, showed a reduced motility in KYSE70 and TE8 cells with the indicated concentrations of CYT-Rx20 compared with that of untreated control (KYSE70: 0.33 ± 0.03 fold vs. 1.00 ± 0.02 fold, *P* <0.001; TE8: 0.39 ± 0.09 fold vs. 1.00 ± 0.10 fold, *P* <0.01) (Fig 2A). In Matrigel-covered transwell assay, KYSE70 and TE8 cells with CYT-Rx20 treatment at the dosages that do not cause obvious cell death showed attenuated cell invasion after 48 h of incubation compared with the control group (KYSE70: 0.26 ± 0.07 fold vs. 1.14 ± 0.16 fold, *P* <0.001; TE8: 0.07 ± 0.01 fold vs. 1.00 ± 0.12 fold, *P* <0.001) (Fig 2B). A crucial event in cancer cell migration and invasion is the epithelial-mesenchymal conversions, and the expression of epithelial to mesenchymal transition (EMT) markers is required for the early steps of metastasis [24,25]. We examined the protein levels of epithelial maker (ZO-1) and mesenchymal markers (N-cadherin, ZEB1, Slug, Snail) in esophageal cancer cells by immunoblotting analysis and found that the level of ZO-1 were markedly increased in KYSE70 and TE8 cells with the indicated concentrations of CYT-Rx20 compared with the control (*P* <0.05 in KYSE70; *P* <0.001 in TE8) (Fig 2C). In contrast, the expression of N-cadherin, ZEB1, Slug, and Snail after CYT-Rx20 incubation was decreased in KYSE70 and TE8 cells compared with control cells (Fig 2C).

### Involvement of PI3K/AKT and STAT3-mediated pathways in CYT-Rx20-suppressed cell viability and migration

**Fig 3:**
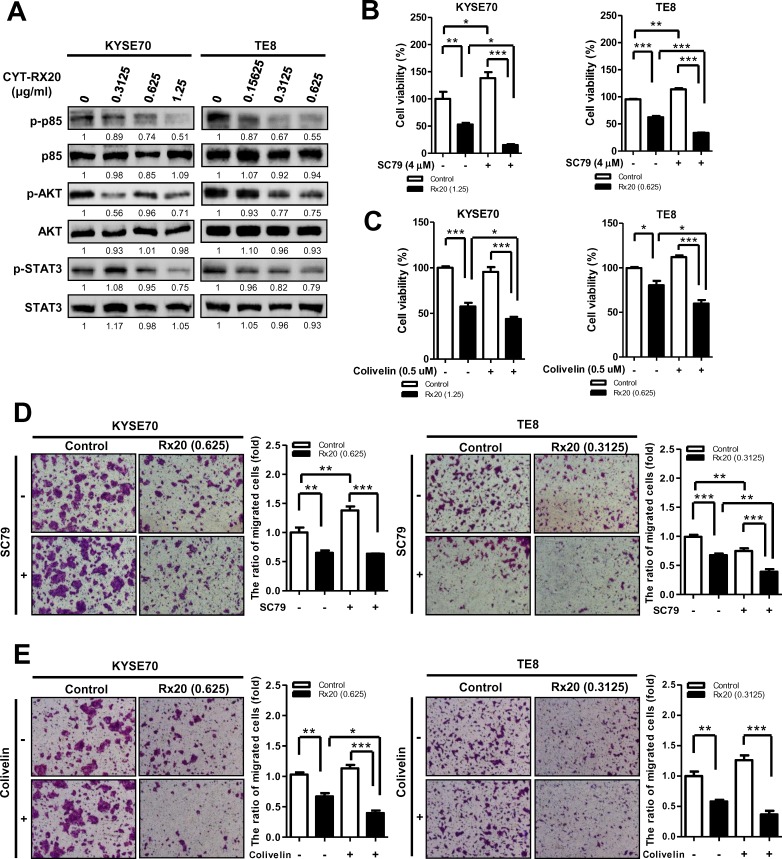
Involvement of PI3K/AKT and STAT3 in CYT-Rx20-inhibited KYSE70 and TE8 cell viability and migration. (A) Dose effect of CYT-Rx20 on p85, AKT, and STAT3 phosphorylation was performed was performed by using immunoblotting. KYSE70 and TE8 were incubated with CYT-Rx20, and cell viability was detected by using XTT colorimetric assay. In parallel cultures, cells were pre-treated with 4 μM SC79 (B) or 0.5 μM colivelin (C) for 1 h and were further co-incubated with CYT-Rx20 for another 72 h. All values are expressed as a percentage of the control group, which is set as 100%. Results are means ± SEM (n = 3–4). *P < 0.05, **P <0.01, ***P <0.001 compared with the indicated group. Using a modified Boyden chamber assay, KYSE70 and TE8 were pre-treated with 4 μM SC79 (D) or 0.5 μM colivelin (E) for 1 h and further treated with CYT-Rx20 for 48 h. Representative photographs are shown with ×40 magnification. Histograms show the average area of migrated cells compared with control. Results are presented as means ± SEM (n = 3). *P < 0.05, **P <0.01, ***P <0.001 compared with the indicated group.

Recent studies have shown that activation of Akt and STAT3 in tumor cells enhances cell migration and growth [26,27]. As shown in Fig 3A, decreased protein expression of phospho-p85, phospho-AKT, and phospho-STAT3 was observed in esophageal cancer cells after CYT-Rx20 treatment, while the total form of p85, AKT, and STAT3 was not significantly altered by CYT-Rx20. To further investigate the role of AKT and STAT3 pathways in CYT-Rx20-reduced cell viability and migration, AKT activator SC79 and STAT3 activator colivelin were applied. CYT-Rx20-treated esophageal cancer cells, when co-treated with AKT activator SC79 or STAT3 activator colivelin, significantly suppressed the viability in KYSE70 and TE8 cells (Fig 3B and 3C). Similar inhibitory effect on cell migration was observed after co-treatment of CYT-Rx20 and the above activators (Fig 3D and 3E).

### CYT-Rx20 inhibited in vitro anchorage-independent cell growth and in vivo xenograft tumor growth

**Fig 4:**
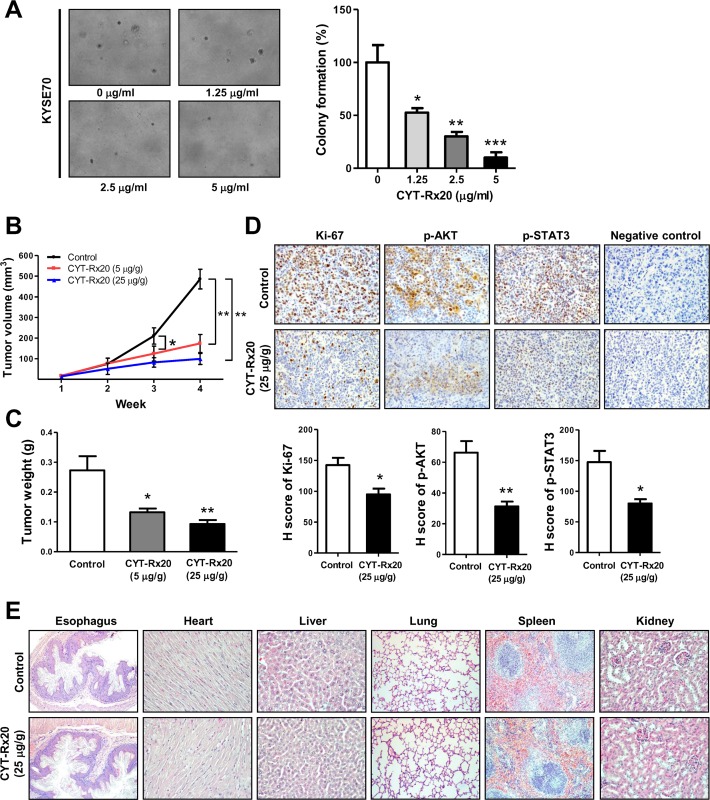
CYT-Rx20 suppressed xenograft tumor growth and decreased phospho-AKT and phospho-STAT3 expression in vivo. (A) KYSE70 cells were treated with the indicated concentrations of CYT-Rx20, followed by evaluation of anchorage-independent colony formation using soft agar assay as described in the Materials and methods section. The representative photographs are shown with ×100 magnification. (B) Female nude mice subcutaneously xenografted with KYSE70 cells were intraperitoneally treated with 0.1% DMSO in normal saline (control), 5 μg/g CYT-Rx20, or 25 μg/g CYT-Rx20 three times per week (n = 10 for each group). Tumor volumes were measured every week for each group and calculated according to the formula of width^2^×length/2. (C) Tumor weight was measured after sacrifice of the mice at the end of the 4-week treatment period. (D) Xenograft tumor tissues were analyzed for the expression of Ki-67, phospho-AKT, and phospho-STAT3 by IHC staining. Negative control was performed in the same procedure but without addition of Ki-67, phospho-AKT, or phospho-STAT3 antibodies. H-score was calculated as the product of percentage of stained cells and intensity of staining. The representative photographs are shown with ×200 magnification. (E) Hematoxylin and eosin (H&E) staining of tissues sections from mouse organs in control and CYT-Rx20 (25 μg/g)-administered mice. The representative photographs are shown with ×100 (Esophagus, Lung, Spleen), ×200 (Heart, Liver, Kidney) magnification. Results are means ± SEM. *P < 0.05, **P <0.01, ***P <0.001 compared with the control group.

The inhibitory effect of CYT-Rx20 on in vitro anchorage-independent tumor growth of esophageal cancer cells was evaluated. The results of soft agar assay showed that CYT-Rx20 (1.25, 2.5 or 5 μg/ml) dose-dependently suppressed the anchorage-independent tumor growth of esophageal cancer cells (Fig 4A). To investigate the anti-cancer activity of CYT-Rx20 in vivo, the nude mice model with subcutaneous xenograft was employed. As shown in Fig 4B, KYSE70 tumor growth was significantly suppressed by CYT-Rx20 treatment in comparison with the untreated control group. After 4 weeks of CYT-Rx20 treatment, the average tumor volumes for the untreated control, CYT-Rx20 (5 μg/g body weight), and CYT-Rx20 (25 μg/g body weight) groups were 486.40 ± 47.59, 174.10 ± 17.83, and 99.13 ± 13.36 mm^3^, respectively (Fig 4B), and the average tumor weights were 0.27 ± 0.05, 0.13 ± 0.01, and 0.09 ± 0.01 g, respectively (Fig 4C). Furthermore, the expression levels of Ki-67, phospho-AKT, and phospho-STAT3 in xenografted tumors were decreased after CYT-Rx20 treatment (Fig 4D). The mice treated with CYT-Rx20 did not show significant changes in the body weights (S2 Fig), biochemical profiles (S1 Table), and histology of esophagus, heart, liver, lung, spleen, and kidney (Fig 4E) compared with the untreated control mice. In addition, no obvious expression of cleaved caspase-3 was observed in the esophageal epithelium of control or CYT-Rx20-administered mice. Of note, mouse lymph node tissue was used as the positive control to assess the levels of cleaved caspase-3 [28,29] and were depicted in S3 Fig.

### CYT-Rx20 inhibited orthotopic esophageal tumor growth and metastasis in vivo

**Fig 5:**
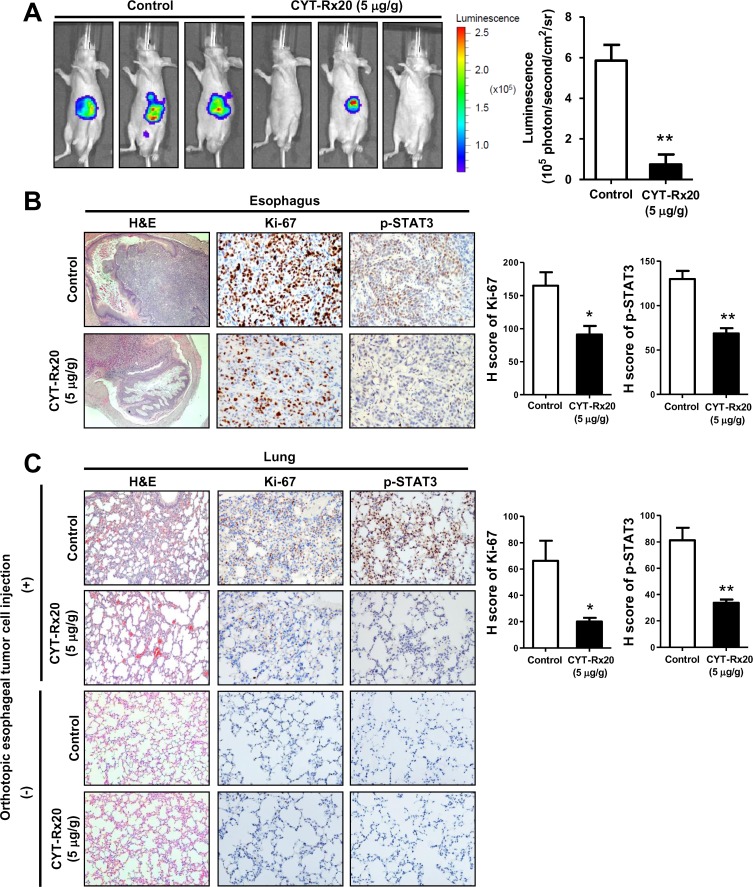
CYT-Rx20 suppressed orthotopic esophageal tumor growth and metastasis in vivo. (A) Luciferase-expressing KYSE70 cells were inoculated into the abdominal esophagus of female nude mice as described in the Materials and methods section. The mice were intraperitoneally injected with 0 μg/g (control) or 5 μg/g of CYT-Rx20 three times a week (n = 4–5 per group). After 4 weeks, the mice were anesthetized and intraperitoneally injected with D-luciferin (150 mg/kg) for detection of bioluminescence by IVIS Spectrum in vivo imaging system. (B) Orthotopic esophageal tumor tissues were analyzed for the expression of Ki-67 and phospho-STAT3 by IHC staining. H-score was calculated as the product of percentage of stained cells and intensity of staining. The representative photographs are shown with ×40 (H&E), ×200 (IHC) magnification. (C) Metastatic lung tumor tissues from mice with or without orthotopic esophageal tumor cell injection were analyzed for the expression of Ki-67 and phospho-STAT3. Histograms show the H-score calculated as the product of percentage of stained cells and intensity of staining. The representative photographs are shown with ×100 (H&E), ×200 (IHC) magnification. Results are means ± SEM. *P < 0.05, **P <0.01 compared with the control group.

To further determine the anti-cancer activity of CYT-Rx20 in vivo, an orthotopic esophageal tumor growth model in nude mice was employed. As shown in Fig 5A, using IVIS Spectrum in vivo imaging system, the mice injected with CYT-Rx20 revealed a significantly lower bioluminescent signal in the abdominal esophagus tissues than the untreated control mice (*P* < 0.01). CYT-Rx20 treatment did not cause significant changes in biochemical parameters of the mice (S2 Table). Furthermore, the expression of Ki-67 and phospho-STAT3 in orthotopic esophageal tumor tissues were decreased after CYT-Rx20 treatment (Fig 5B), in agreement with our in vitro observation. Finally, similar inhibitory effect of CYT-Rx20 on the expression of Ki-67 and phospho-STAT3 was observed in the metastatic lung tumor tissues (Fig 5C).

## Discussion

The β-nitrostyrene family compounds possess diverse biological activities, including induction of cytotoxicity in cancer cells [13,30]. Previous report demonstrated that CYT-Rx20 inhibited breast cancer cell proliferation and tumorigenesis via increase of ROS-mediated MEK/ERK signaling pathway [18]. In this study, we demonstrated for the first time that CYT-Rx20 suppressed the PI3K/AKT and STAT3 signaling pathways and inhibited esophageal cancer cell viability and migration. These results suggested possible tissue-specific anti-cancer activities of CYT-Rx20.

Activation of PI3K/AKT signaling pathway associates with a variety of cellular functions including cell growth, survival, vascular remodeling, angiogenesis, and metastasis [31,32]. Accumulating evidence suggests that suppression of AKT activity is a feasible approach for inhibition of tumor progression [33–35]. In this study, we observed an activation of caspase cascade and PARP cleavage after CYT-Rx20 treatment (Fig 1E), indicating an increased cytotoxicity in esophageal cancer cells. Besides, we also found a reduced migration and invasion in KYSE70 and TE8 cells after CYT-Rx20 treatment (Fig 2), associated with a decreased expression of phospho-p85 and phospho-AKT in esophageal cancer cells (Fig 3A). Therefore, our results provide evidence for the potential application of CYT-Rx20 in esophageal cancer treatment by targeting AKT.

Other than PI3K/AKT signaling network [26], previous reports also demonstrated an essential role of STAT3 [36,37] and its regulation by PI3K/mTOR signaling [38,39] in tumor progression. While a previous study reported the STAT3 mediates PI3K/AKT activation upon interleukin-6 treatment [40,41], our study showed a decreased protein expression of phospho-p85, phospho-AKT, and phospho-STAT3 in esophageal cancer cells after CYT-Rx20 treatment (Fig 3A). Furthermore, pre-treatment of STAT3 activator colivelin followed by CYT-Rx20 decreased the expression of phospho-STAT3, but not phospho-AKT, to a larger extent (**data not shown**), suggesting that STAT3 functions downstream of AKT. While further efforts are required to resolve this dispute, differential activity of CYT-Rx20 and interleukin-6 on cancer cells can not be ruled out.

While CYT-Rx20 treatment inhibited esophageal cancer cell viability, co-treatment with SC79 or colivelin led to a greater reduction in esophageal cancer cell viability, compared with cells only treated with CYT-Rx20 (Fig 3B and 3C). In addition, esophageal cancer cells co-treated with CYT-Rx20 and SC79 (or colivelin) resulted in enhanced decrease in cell migration (Fig 3D and 3E). Oncogene addiction is a phenomenon regarding the dependence of cancer cells on a single activated oncogenic protein or pathway for proliferation and survival [42–45]. For example, the Bcr-Abl inhibitor imatinib selectively induced cytotoxicity in Bcr-Abl-transfected leukemic cells but not Bcr-Abl-negative parental cells [46]. Also, Bcr-Abl mutant-transfected leukemic cells are resistance to the killing by imatinib compared with Bcr-Abl-positive parental cells, suggesting that the cancer cells rely on Bcr-Abl for survival [47]. In another study, treatment of c-MYC-expressing myeloma cells with the 10058-F4 compound, an inhibitor of MYC-MAX heterodimerization, resulted in rapid apoptosis, suggesting that there is an addiction to c-MYC for survival in these cancer cells [48]. There are different types of oncogene addiction according to cellular activities. For example, K-Ras-mediated cancer growth depends on the elevated levels of autophagy (referred to autophagy addiction) [49]. Hall *et al*. [50] demonstrated that malignant melanoma cells were addicted to ^V600E^BRAF-driven glycolysis for efficient ATP production. Furthermore, our previous data indicated that, while overexpression of Id1 promoted the lung cancer cell growth, treatment of paclitaxel and cisplatin caused a greater reduction on cancer cell growth in the Id1-overexpressing cells, suggesting that lung cancer cells with high Id1 levels may depend on Id1 for survival [51]. Therefore, our current findings suggest that high cellular AKT or STAT3 expression leads esophageal cancer cells to rely on AKT or STAT3 for survival as a result of oncogene addiction.

In this study, the anti-cancer activity of CYT-Rx20 was evaluated in vivo. CYT-Rx20 suppressed xenografted (Fig 4B) and orthotopic (Fig 5A) esophageal tumor growth along with decreased expression of Ki-67, phospho-AKT, and phospho-STAT3 in tumor tissues (Figs 4D and 5B). In addition, we found no obvious impairment of renal or liver functions (S1 and S2 Tables), nor did it cause histological changes in major organs of nude mice including esophagus (Fig 4E), rendering CYT-Rx20 a potentially low side-effect anti-esophageal cancer agent.

Alterations in cell migration, invasion, EMT, and production of matrix metalloproteinase are associated with diverse pathologic changes, such as angiogenesis, restenosis in grafted or injured vessels, and cancer [52–54]. CYT-Rx20 treatment rendered esophageal tumor cells the disability to invade and migrate (Fig 2A and 2B). In agreement with this finding, CYT-Rx20 treatment in an orthotopic esophageal tumor model significantly decreased lung metastasis in mice (Fig 5A and 5C). Furthermore, CYT-Rx20 treatment had an inhibitory effect on cancer cell migration and invasion through activation of epithelial maker (ZO-1) and inactivation of mesenchymal markers (N-cadherin, ZEB1, Slug, Snail) (Fig 2C). EMT is a reversible multistep process defined by the loss of epithelial phenotype and the acquisition of mesenchymal characteristics [55]. For inhibition of cancer cell metastasis, an induction of cell-cell adhesion (characterized by an increase of epithelial cell adhesion proteins including the tight junction proteins, ZO-1 and claudin-1) is required [56]. Lennon *et al*. [57] demonstrated that STAT3 inhibitor Stattic decreased the opioid and EGF-induced human lung cancer cell proliferation, migration, and EMT transformation (as determined by loss of mesenchymal proteins such as vimentin and the acquisition of epithelial markers including claudin-1). It was also reported that JAK2/STAT3 inhibitor AG490 regulates the activity of EGF on mesenchymal (vimentin and fibronectin) or epithelial (E-cadherin, ZO-1) markers [58]. Park *et al*. [59] showed that Sorafenib, a multikinase inhibitor, blocked phospho-STAT3 and then suppressed Epstein-Barr virus-transformed adult retinal pigment epithelial cells through blocking the upregulation of vimentin, Snail, and ZEB1 as well as recovering the expression of ZO-1, claudin-1, and E-cadherin. Inactivation of STAT3-EMT or AKT-EMT axis has been shown to inhibit cancer cell migration [57]. In agreement with this report, we observed inactivation of STAT3-EMT and AKT-EMT axes is responsible for the inhibition of migration and invasion by CYT-Rx20 *in vitro* and *in vivo* (Figs 2 and 5C). To the best of our knowledge, this is the first report showing the anti-metastasis activity of CYT-Rx20 on esophageal cancer cells.

In conclusion, CYT-Rx20 inhibited esophageal cancer cell viability and metastasis *in vitro* and *in vivo* through down-regulation of AKT and STAT3 activities. Further pre-clinical studies are required to confirm its anti-cancer activity for esophageal cancer.

## Supporting Information

S1 Fig Effect of CYT-Rx20 on cytotoxicity in normal esophageal squamous cells. Normal human esophageal squamous cell line HET-1A was treated with various concentrations of CYT-Rx20 for 48 h and assessed by XTT colorimetric assay. Results are repeated three times with five replicate wells per CYT-Rx20 concentration in each experiment and presented as means ± SEM.
(TIF)

S2 Fig Effect of CYT-Rx20 on body weight of xenograft tumor mice. Female nude mice subcutaneously xenografted with KYSE70 cells were intraperitoneally treated with normal saline (control), 5 μg/g CYT-Rx20, or 25 μg/g CYT-Rx20 three times per week (n = 10 for each group). Body weights of mice were measured every week for each group. Results are presented as means ± SEM.
(TIF)

S3 Fig Detection of cleaved caspase-3 in esophageal tissues of control and CYT-Rx20 -administered mice. IHC staining for cleaved caspase-3 in esophageal tissue sections in control and CYT-Rx20 (25 μg/g)-administered mice. Mouse axillary lymph node tissues were used as the positive control to assess the levels of cleaved caspase-3. The representative photographs are shown with ×200 (Esophagus), ×400 (lymph node) magnification.
(TIF)

S1 Table Biochemical profiles of the nude mice after treatment with CYT-Rx20 for 4 weeks. (DOC)

S2 Table Biochemical profiles of the orthotopic esophageal cancer mice after treatment with CYT-Rx20 for 4 weeks. (DOC)
