# Supplementary figures and images for: The Synthetic β-Nitrostyrene Derivative CYT-Rx20 Inhibits Esophageal Tumor Growth and Metastasis via PI3K/AKT and STAT3 Pathways
Source: PLoS One. 2016 Nov 22;11(11):e0166453. doi: 10.1371/journal.pone.0166453 (PMC5119777; doi:10.1371/journal.pone.0166453)

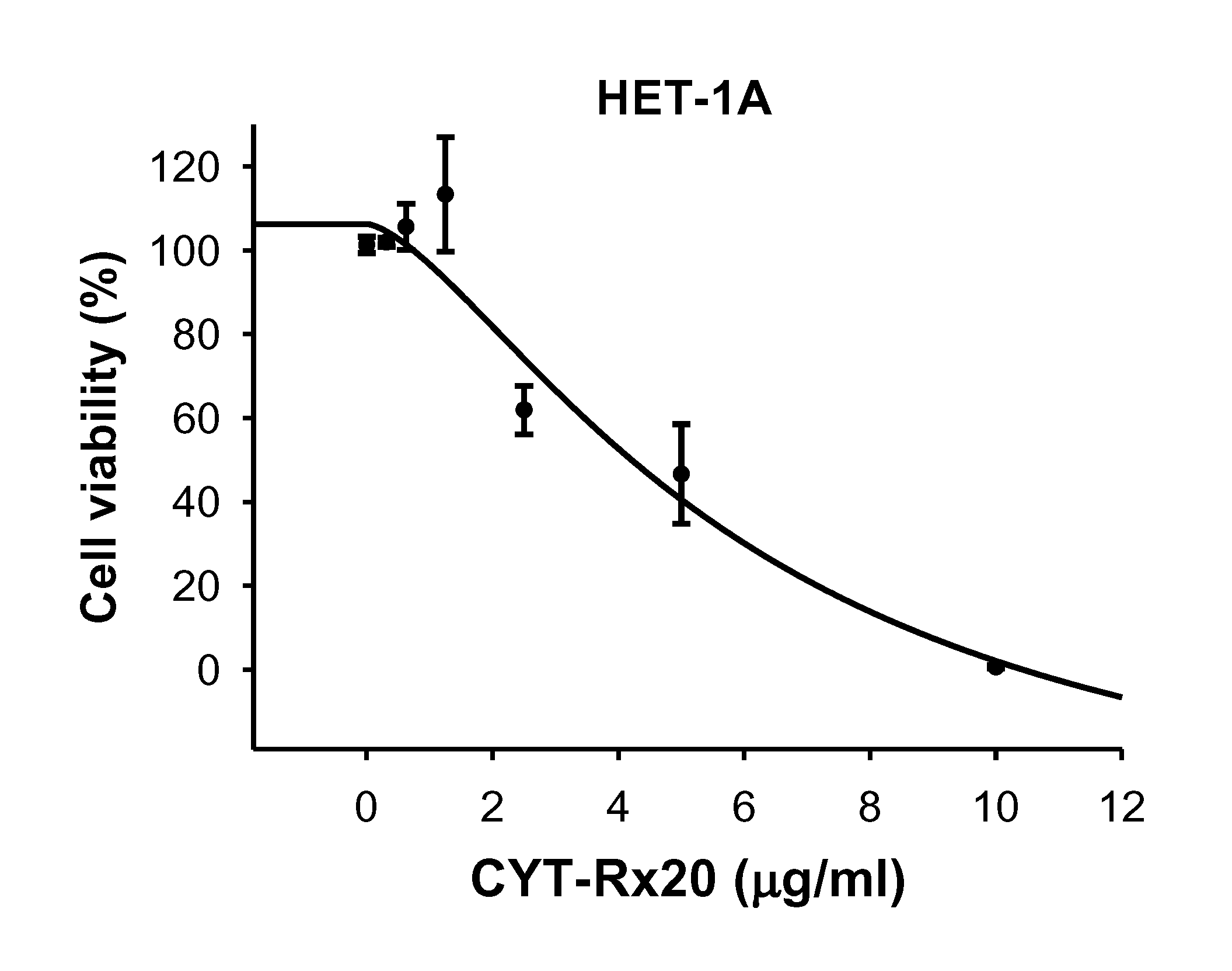

Supplement: S1 Fig — Normal human esophageal squamous cell line HET-1A was treated with various concentrations of CYT-Rx20 for 48 h and assessed by XTT colorimetric assay. Results are repeated three times with five replicate wells per CYT-Rx20 concentration in each experiment and presented as means ± SEM. (TIF) [file pone.0166453.s001.tif]

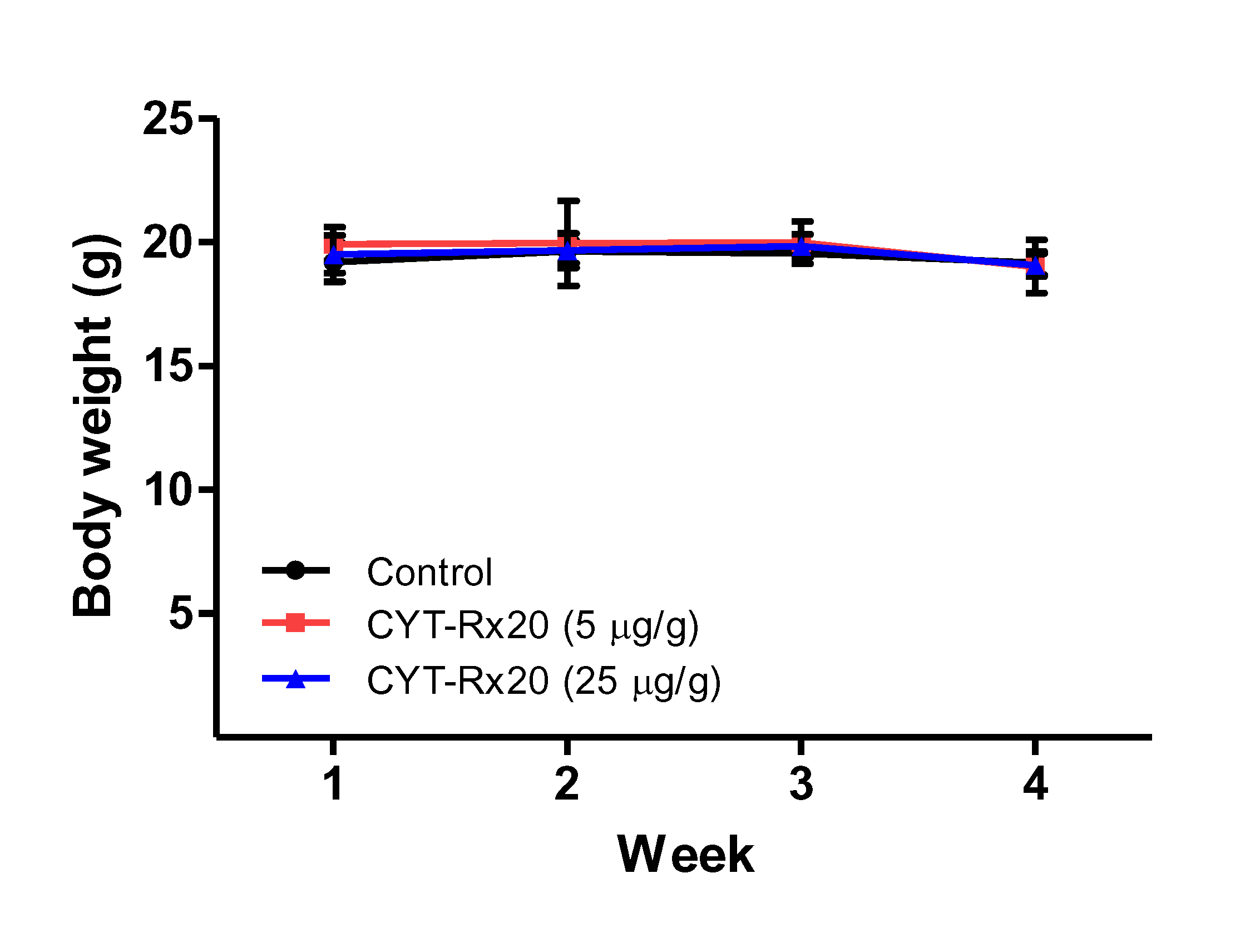

Supplement: S2 Fig — Female nude mice subcutaneously xenografted with KYSE70 cells were intraperitoneally treated with normal saline (control), 5 μg/g CYT-Rx20, or 25 μg/g CYT-Rx20 three times per week (n = 10 for each group). Body weights of mice were measured every week for each group. Results are presented as means ± SEM. (TIF) [file pone.0166453.s002.tif]

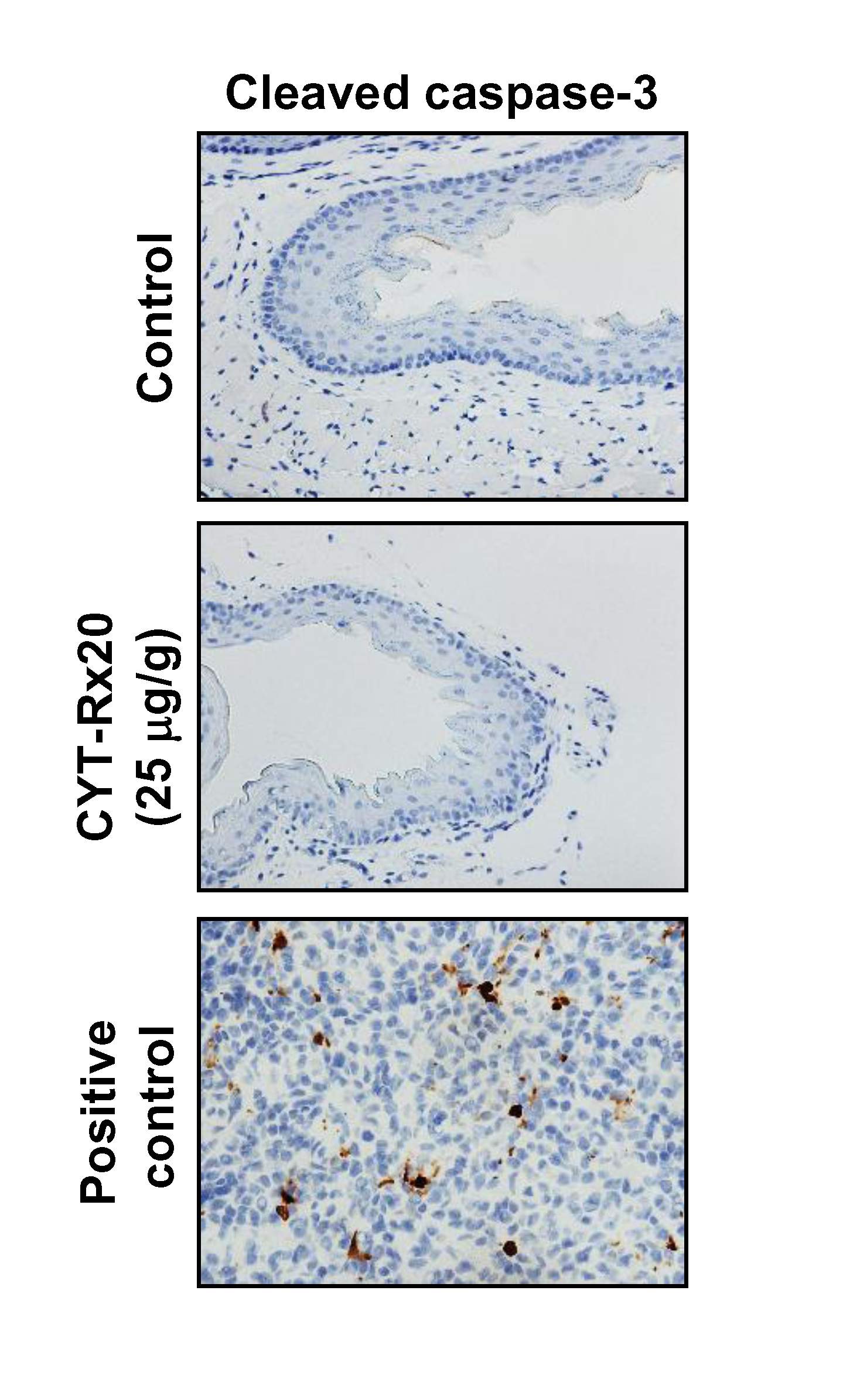

Supplement: S3 Fig — IHC staining for cleaved caspase-3 in esophageal tissue sections in control and CYT-Rx20 (25 μg/g)-administered mice. Mouse axillary lymph node tissues were used as the positive control to assess the levels of cleaved caspase-3. The representative photographs are shown with ×200 (Esophagus), ×400 (lymph node) magnification. (TIF) [file pone.0166453.s003.tif]
