# Supplementary material for: The Synthetic β-Nitrostyrene Derivative CYT-Rx20 Inhibits Esophageal Tumor Growth and Metastasis via PI3K/AKT and STAT3 Pathways
Source: PLoS One. 2016 Nov 22;11(11):e0166453. doi: 10.1371/journal.pone.0166453 (PMC5119777; doi:10.1371/journal.pone.0166453)
Supplement: S1 Table — (DOC) [file pone.0166453.s004.doc]

**S1 Table.** **Biochemical profiles of the nude mice after treatment with CYT-Rx20 for 4 weeks.**

| Variables | Control | CYT-Rx20^a^  (5 μg/g) | CYT-Rx20^a^  (25 μg/g) |
| --- | --- | --- | --- |
| GOT (U/l) | 141.75 ± 54.05 | 107.60 ± 17.64 | 113.80 ± 60.11 |
| GPT (U/l) | 49.25 ± 8.54 | 44.00 ± 6.96 | 43.80 ± 7.73 |
| BUN (mg/dl) | 29.18 ± 3.95 | 27.10 ± 2.26 | 24.98 ± 5.05 |
| Creatinine (mg/dl) | 0.18 ± 0.01 | 0.17 ± 0.04 | 0.16 ± 0.02 |

^a^Data were presented as mean ± SD.
