# Supplementary material for: The Synthetic β-Nitrostyrene Derivative CYT-Rx20 Inhibits Esophageal Tumor Growth and Metastasis via PI3K/AKT and STAT3 Pathways
Source: PLoS One. 2016 Nov 22;11(11):e0166453. doi: 10.1371/journal.pone.0166453 (PMC5119777; doi:10.1371/journal.pone.0166453)
Supplement: S2 Table — (DOC) [file pone.0166453.s005.doc]

**S2 Table.** **Biochemical profiles of the orthotopic esophageal cancer mice after treatment with CYT-Rx20 for 4 weeks.**

| Variables | Control | CYT-Rx20^a^  (5 μg/g) |
| --- | --- | --- |
| GOT (U/l) | 123.80 ± 59.84 | 81.75 ± 18.55 |
| GPT (U/l) | 41.40 ± 12.44 | 31.88 ± 6.03 |
| BUN (mg/dl) | 32.05 ± 9.37 | 31.67 ± 9.12 |
| Creatinine (mg/dl) | 0.47 ± 0.39 | 0.15 ± 0.02 |

^a^Data were presented as mean ± SD.
